# Effects of restrictions to Income Support on health of lone mothers in the UK: a natural experiment study

**DOI:** 10.1016/S2468-2667(18)30109-9

**Published:** 2018-07-02

**Authors:** Srinivasa Vittal Katikireddi, Oarabile R Molaodi, Marcia Gibson, Ruth Dundas, Peter Craig

**Affiliations:** a Medical Research Council/Chief Scientist Office Social and Public Health Sciences Unit, University of Glasgow, Glasgow, UK

## Abstract

**Background:**

In the UK, lone parents must seek work as a condition of receiving welfare benefits once their youngest child reaches a certain age. Since 2008, the lower age limit at which these Lone Parent Obligations (LPO) apply has been reduced in steps. We used data from a nationally representative, longitudinal, household panel study to analyse the health effects of increased welfare conditionality under LPO.

**Methods:**

From the Understanding Society survey, we used data for lone mothers who were newly exposed to LPO when the age cutoff was reduced from 7 to 5 years in 2012 (intervention group 1) and from 10 to 7 years in 2010 (intervention group 2), as well as lone mothers who remained unexposed (control group 1) or continuously exposed (control group 2) at those times. We did difference-in-difference analyses that controlled for differences in the fixed characteristics of participants in the intervention and control groups to estimate the effect of exposure to conditionality on the health of lone mothers. Our primary outcome was the difference in change over time between the intervention and control groups in scores on the Mental Component Summary (MCS) of the 12-item Short-Form Health Survey (SF-12).

**Findings:**

The mental health of lone mothers declined in the intervention groups compared with the control groups. For intervention group 1, scores on the MCS decreased by 1·39 (95% CI −1·29 to 4·08) compared with control group 1 and by 2·29 (0·00 to 4·57) compared with control group 2. For intervention group 2, MCS scores decreased by 2·45 (−0·57 to 5·48) compared with control group 1 and by 1·28 (−1·45 to 4·00) compared with control group 2. When pooling the two intervention groups, scores on the MCS decreased by 2·13 (0·10 to 4·17) compared with control group 1 and 2·21 (0·30 to 4·13) compared with control group 2.

**Interpretation:**

Stringent conditions for receiving welfare benefits are increasingly common in high-income countries. Our results suggest that requiring lone parents with school-age children toseek work as a condition of receiving welfare benefits adversely affects their mental health.

**Funding:**

UK Medical Research Council, Scottish Government Chief Scientist Office, and National Health Service Research Scotland.

## Introduction

Lone-parent families tend to have poorer health, greater poverty, and lower employment than do two-parent families.^1^, ^2^, ^3^ The proportion of families headed by a lone parent (defined as a parent that is single, divorced, or widowed) has increased in many high-income countries,^4^ with 25% of all families with dependant children in the UK now headed by a lone parent.^5^ Governments have attempted to reduce the associations between single parenthood, poverty, and poor health by increasing lone parents' participation in paid work and reducing the number receiving welfare benefits. One such welfare-to-work measure requires claimants to be available for work and to demonstrate active job seeking. Known as conditionality, such measures have become increasingly common in social security systems worldwide, including in Australia, Canada, New Zealand, and Norway.

For the period covered by our study (2009–13), Income Support was the primary form of social security benefit payable to lone parents in the UK who were not in work and had no other source of income. The amount was intended to cover basic subsistence only: in 2009, £73·10 per week was payable to lone parents aged 18 years or older and £57·90 to those aged 16 or 17 years. Before 2008, lone parents whose youngest child was younger than 16 years were eligible to receive Income Support without having to show that they were available for and actively seeking work. On Nov, 25, 2008, the UK Government introduced conditionality for lone parents receiving Income Support for children younger than the minimum school leaving age, requiring them to be available for work for a minimum of 16 h per week when their youngest child reached age 12 years.^6^ The age cutoff was further reduced to 10 years from Nov 24, 2009, to 7 years from Oct 26, 2010, and to 5 years from May, 2012. Outside the range of our study, the age threshold was then reduced to 3 years in 2017.

Under Lone Parent Obligations (LPO), lone parents are transferred from Income Support to Jobseeker's Allowance once their youngest child reaches the age threshold. Receipt of Jobseeker's Allowance requires lone parents to be available for and actively seeking work within their children's school hours. Jobcentre Plus advisers can require claimants to attend training courses, apply for a certain number of jobs per week, or spend a specified number of hours per week looking for work. Failure to comply with these requirements might lead to sanctions, involving cessation of Jobseeker's Allowance for a minimum of 4 weeks but up to a maximum of 3 years. The annual rate of sanctioning for lone parents on Jobseeker's Allowance increased from 3% in 2008–09 to 14% in 2012–13, with the majority of these being low-level sanctions that lasted for 4 weeks.^7^ Lone parent flexibilities that allow lone parents to restrict hours of work to children's normal school hours and exempt them from work requirements during school holidays are sometimes applied if the Jobcentre Plus adviser accepts that appropriate or affordable child care is not available. During the period of the study (2009–13), if a lone parent moved into paid work of 16 h or more per week, they became eligible for Working Tax Credit and were then no longer subject to job search requirements.

**Research in context**

**Evidence before this study**

We searched MEDLINE and EconPapers up to June 30, 2017, using the keywords “lone parent”, “welfare reform”, and “health”. All quantitative evidence available on the health effects of mandatory work requirements on lone parents comes from the USA. A Cochrane systematic review of randomised controlled trials of mandatory welfare-to-work interventions, primarily implemented in North America, found that although the very small effects on health were largely positive, they were unlikely to be clinically meaningful. Analyses of data from the samples in two of the included studies 15–17 years after randomisation reported small negative effects on mortality. Econometric studies done soon after the implementation of welfare reform in the USA reported mixed effects on adult health. More recently, studies using representative data that capture the longer-term effects of welfare reform in the USA have shown small but consistently negative effects on health behaviours, measures of mental health, self-reported health, and self-reported disability. Evidence from a systematic review of qualitative research and a subsequent qualitative study indicates that respondents frequently link work requirements to stress, depression, and anxiety.

**Added value of this study**

To our knowledge, our study is the first outside of the USA to use robust quasi-experimental methods to investigate the health effects of mandatory job-seeking requirements for lone mothers. We used nationally representative data from the largest household panel study in the UK for the period of 2009–13. We examined two changes in the child age cutoff for Income Support that determines exposure to the job-seeking requirement, and identified a range of intervention and control groups to check the consistency of our findings. We found that mental health was consistently worse among lone mothers newly exposed to the employment requirements because of a change in the child age cutoff compared with lone mothers who were unexposed or continuously exposed.

**Implications of all the available evidence**

Mandatory employment requirements for lone parents have become widespread in high-income countries. The available evidence suggests that they are associated with negative effects on lone parents' mental health. Although these effects are typically small at the individual level, they affect large numbers of families and might have important implications for population health. Given that lone parents experience relatively poor health compared with parents in two-parent families, any policy that might further harm their health should be carefully evaluated and mitigating measures implemented if harms are identified. Further research should focus on the longer-term effects of mandatory employment requirements and on the effects of reducing the child age threshold to 3 years.

The effect of these changes on the health of lone parents is unknown. In its equality impact assessments of the 2017 reduction in the child age threshold to 3 years, the UK Department of Work and Pensions stated that there would be positive effects on wellbeing.^8^ Studies^9^ of welfare-to-work interventions in the USA found only small positive effects on health and economic outcomes.

There are a number of mechanisms through which LPO might affect the health of lone parents. On the one hand, becoming employed or participating in training as a result of the intervention could improve health and potentially narrow health inequalities.^10^, ^11^ On the other hand, conflicts between work or training requirements and child-care availability could increase stress and role strain.^10^ Financial sanctions for non-compliance might lead to increased stress and financial strain. The health benefits of employment are contingent upon job quality.^12^, ^13^ A move into work might increase income, but if the job is of poor quality or perceived as insecure, it might have no or very little benefit.

In this study, we aimed to investigate the effects of LPO on the physical and mental health of lone mothers in the UK using data from the Understanding Society survey.

## Methods

### Study design

Understanding Society is a nationally representative, longitudinal, household panel study based on a stratified random probability sample of about 40 000 households from the four UK countries.^14^ Data are predominantly collected by trained interviewers using face-to-face surveys, with all adults (aged ≥16 years) in the chosen households invited to participate. Each data collection wave lasts 24 months, but participants are followed up annually, with interviews scheduled at the same time each year.

We used changes in the age cutoff for Income Support to identify groups of lone mothers who were newly exposed to LPO, and compared changes in their health before and after the policy change with changes in health among lone mothers whose exposure status was unchanged. Exposure status was defined on the basis of the age of the youngest child at 1 year before the change in age eligibility (baseline) to allow for an intention-to-treat effect to be estimated (ie, regardless of change in relationship status or pregnancy that might occur as a result of the policy and alter exposure to LPO). Because of the small number of lone fathers in the UK, we restricted the primary analysis to lone mothers. We excluded lone parents who were receiving sickness or disability benefits at baseline because they were not subject to job-seeking requirements.

**Figure 1:**
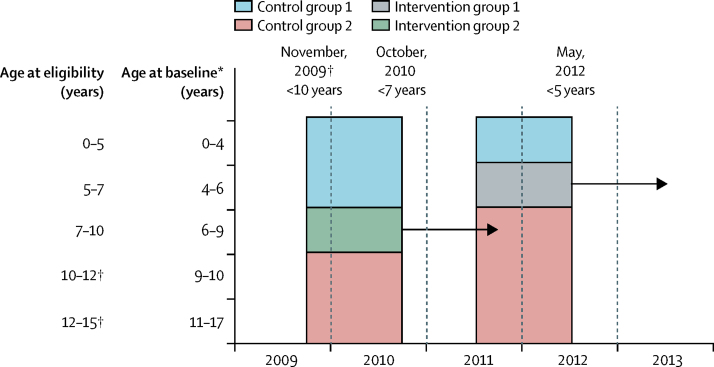
Schema for defining study groups Participants in intervention group 1 were newly exposed to LPO when the age cutoff was changed from 7 to 5 years in 2012, those in intervention group 2 were newly exposed when the age cutoff was changed from 10 to 7 years in 2010, those in control group 1 were unexposed, and those in control group 2 were continuously exposed. The arrows indicate follow-up (ie, the period from the date of policy change to 1 year after the change), and the horizontal lines indicate exposure groups. LPO=Lone Parent Obligations. *Exposure status was based on age of youngest child at 1 year before the change in the age eligibility cutoff for LPO. †Baseline measures for these groups could not be found within Understanding Society, and so they were not included in the analysis.

The stepwise lowering of the age cutoff allowed the identification of two intervention groups within our dataset with pre-intervention and post-intervention measures (figure 1). Intervention group 1 comprised lone mothers who were exposed to LPO after the May, 2012, change in the age eligibility cutoff from 7 to 5 years. This group included lone mothers receiving Income Support whose youngest child was aged 4–6 years at baseline. Intervention group 2 comprised lone mothers who were exposed to LPO after the October, 2010, change in the cutoff from 10 to 7 years. This group included lone mothers receiving Income Support whose youngest child was aged 6–9 years at baseline. For each intervention group, we identified two control groups. The control groups for intervention 1 were defined by identifying lone mothers whose youngest child at baseline was aged 0–3 years (control group 1; always unexposed) or 7–14 years (control group 2; always exposed). Similarly, the control groups for intervention 2 were defined by identifying lone mothers whose youngest child at baseline was aged 0–5 years (control group 1) or 10–14 years (control group 2).

This study follows the recommendations of the UK Medical Research Council guidelines on the evaluation of natural experiments,^15^ which emphasise the value of multiple testing to address the range of possible biases that might affect causal inferences drawn from observational data. The Understanding Society study was approved by the University of Essex Ethics Committee and the National Research Ethics Service. No additional ethical approval was required for this secondary data analysis. Participants had provided written informed consent for taking part in the survey.

### Health outcome measures

Our primary outcome was the difference in change over time between the intervention and control groups in lone mothers' scores on the Mental Component Summary (MCS) of the 12-item Short-Form Health Survey (SF-12). SF-12 is a validated measure of health-related quality of life, which has a range of 0–100 (where higher values indicate better health), a mean of 50, and a SD of 10. The MCS has been shown to have acceptable validity in detecting both recent and active depression in the general population.^16^ We analysed two secondary outcomes: the Physical Health Summary score of the SF-12 and self-rated general health on a five-point Likert scale. Given the time-lag between changes in the social determinants of health and effects on physical health, we anticipated these outcomes to be less sensitive in the short term than the primary outcome to the effects of policy change.

### Statistical analysis

To provide a robust estimate of the effect of LPO on health, we did a difference-in-difference analysis using a longitudinal individual-level dataset by fitting an individual fixed-effects linear regression model including an interaction term between follow-up and intervention group (appendix).^17^ This method allowed comparison of changes in health in the intervention groups with changes in health in the control groups, thereby accounting for fixed characteristics of the groups and for time-varying trends under the assumption that they affect the intervention and control groups similarly. The use of two control groups per intervention group reduced the risk that this common trends assumption was violated, such as by post-partum changes in the mental health of mothers with very young children.

The differences-in-differences analysis should account for confounders that are not time varying. However, given the macroeconomic changes occurring during the study period, coupled with the broader package of austerity policies in the UK, we included prespecified covariates in our analysis to reduce the risk of time-varying confounding. Covariates were maternal age, number of children (one *vs* two, *vs* three or more), and highest educational attainment (none or lower secondary *vs* upper secondary *vs* tertiary). Although, generally, lone parents in the UK experience socioeconomic disadvantage, not all lone parents experience it to the same extent and, therefore, we explored the potential association with socioeconomic inequalities among lone parents by additionally testing for an interaction with educational attainment.

Our primary objective was a comparison of intervention group 1 (lone mothers exposed to LPO when the child age threshold was reduced from 7 to 5 years) with its control groups. In addition to the separate comparisons of intervention groups 1 and 2, we calculated a pooled effect in which the two intervention groups were combined on the assumption that the effect size was similar for both groups. As in the primary analysis, we first compared the combined intervention group with the combined unexposed control groups and then with the combined exposed control groups.

To investigate whether the effect for the combined intervention group differed by educational attainment, we modified the fixed-effect linear regression model through addition of an interaction term between educational attainment and the difference-in-difference estimator, while accounting for differential effect sizes by inclusion of a dummy variable for intervention period. We assigned lowest educational attainment as the reference category; therefore, a positive coefficient indicated that the intervention widened health inequalities, whereas a negative coefficient indicated a narrowing of health inequalities.

To reduce bias arising from survey item non-response, we did multiple imputation with chained equations (20 rounds). The primary analysis used the imputed weighted data. We repeated the analyses for the two secondary outcomes.

We did several analyses to check the robustness of our findings. First, we excluded from control group 1 lone parents whose youngest child was younger than 1 year at baseline. Second, we included both lone fathers and lone mothers. Third, we did complete-case analyses including only participants with no missing data. Fourth, we repeated our analyses without using longitudinal weights. Although weighting can address potential bias arising from attrition, missing data from covariates used to calculate the weights result in exclusion of part of the sample. Fifth, we fitted models with control group 2 restricted to mothers whose youngest child was within the 3 year age range of 7–9 years (for intervention group 1) or 10–12 years (for intervention group 2). Sixth, we repeated the analysis for the combined intervention group using dummies rather than continuous variables for maternal age and number of children. Finally, we tested the assumption of common trends by plotting trends in the outcomes for intervention group 1 and its two control groups across two preintervention survey waves and one post-intervention survey wave (appendix), and by fitting difference-in-difference models with maternal demographic characteristics (age and education) as outcomes. All statistical analyses were done with Stata version 14.

### Role of the funding source

The funders of the study had no role in study design, data collection, data analysis, data interpretation, or writing of the report. The corresponding author had full access to all of the data in the study and had final responsibility for the decision to submit for publication.

## Results

**Figure 2:**
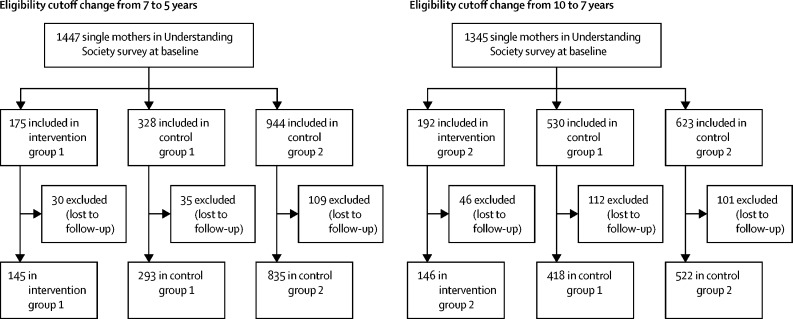
Flowchart of study participants

2359 lone mothers who were included in the Understanding Society survey between 2009 and 2013 were followed up and included in this analysis (figure 2). 145 lone mothers were included in intervention group 1 (LPO age cutoff changed from 7 to 5 years) and 146 in intervention group 2 (based on a change in age cutoff from 10 to 7 years). For intervention group 1, control group 1 included 293 lone mothers and control group 2 included 835. For intervention group 2, control group 1 included 418 mothers and control group 2 included 522. Table 1 presents the characteristics of all participants who had data available at baseline and follow-up. Some differences were observed in maternal age, education level, and number of children between the intervention and control groups. As would be expected, mothers in control group 1 (with younger children) were slightly younger and those in control group 2 (with older children) were slightly older than mothers in the intervention groups. The timing of data collection was similar between the intervention groups, with a median follow-up after policy change of 178 days (IQR 117–298) in intervention group 1 and 172 days (113–263) in intervention group 2.

**Table 1:** Characteristics of study participants (complete-case analysis)

		**Intervention group 1 (n=94)**		**Control group 1 (n=187)**		**Control group 2 (n=571)**		**Intervention group 2 (n=83)**		**Control group 1 (n=252)**		**Control group 2 (n=284)**	
		Baseline	Follow-up	Baseline	Follow-up	Baseline	Follow-up	Baseline	Follow-up	Baseline	Follow-up	Baseline	Follow-up
Maternal age (years)		33·6 (6·6)	34·7 (6·7)	27·6 (6·9)	28·7 (6·8)	40·1 (6·7)	41·2 (6·7)	35·2 (7·0)	36·2 (7·0)	29·1 (7·1)	30·1 (7·1)	41·8 (5·9)	42·8 (5·9)
Number of children													
	One	40 (43%)	38 (40%)	67 (36%)	65 (35%)	331 (58%)	367 (64%)	32 (39%)	30 (36%)	90 (36%)	84 (33%)	174 (61%)	203 (71%)
	Two	36 (38%)	35 (37%)	72 (39%)	72 (39%)	192 (34%)	165 (29%)	35 (42%)	37 (45%)	96 (38%)	105 (42%)	99 (35%)	71 (25%)
	Three or more	18 (19%)	21 (22%)	48 (26%)	50 (27%)	48 (8%)	39 (7%)	16 (19%)	16 (19%)	66 (26%)	63 (25%)	11 (4%)	10 (4%)
Highest education													
	None or lower secondary	66 (70%)	66 (70%)	134 (72%)	134 (72%)	315 (55%)	315 (55%)	61 (73%)	61 (73%)	201 (80%)	201 (80%)	136 (48%)	136 (48%)
	Upper secondary	9 (10%)	9 (10%)	17 (9%)	17 (9%)	47 (8%)	47 (8%)	10 (12%)	10 (12%)	13 (5%)	13 (5%)	35 (12%)	35 (12%)
	Tertiary	19 (20%)	19 (20%)	36 (19%)	36 (19%)	209 (37%)	209 (37%)	12 (14%)	12 (14%)	38 (15%)	38 (15%)	113 (40%)	113 (40%)
Time from policy change to data collection (days)													
	Mean (SD)	208·8 (96·1)	207·4 (101·7)	204·7 (102·7)	221·0 (104·5)	200·0 (103·6)	218·3 (103·4)	185·1 (93·0)	180·5 (92·0)	194·6 (99·4)	171·7 (97·2)	192·1 (95·7)	173·9 (94·0)
	Median (IQR)	219 (127–280)	178 (117–298)	219 (127–311)	208 (147–298)	188 (96–280)	208 (117–298)	193 (102–252)	172 (113–263)	193 (102–283)	172 (82–263)	193 (102–252)	172 (113–263)
SF-12 Physical Health Summary score		50·0 (10·8)	49·8 (10·0)	51·8 (8·7)	51·6 (9·4)	51·4 (10·4)	45·5 (11·6)	51·8 (9·2)	50·9 (10·9)	52·3 (9·3)	50·9 (9·4)	50·2 (11·2)	50·0 (11·2)
SF-12 Mental Component Summary score		43·5 (12·0)	41·1 (12·4)	46·0 (10·2)	44·7 (11·6)	45·5 (11·6)	45·2 (11·5)	45·6 (11·2)	41·9 (14·9)	45·0 (12·2)	46·4 (11·0)	46·5 (10·9)	46·2 (11·4)
Self-rated health		2·8 (1·1)	3·0 (1·1)	2·6 (0·98)	2·8 (1·1)	2·6 (1·1)	2·7 (1·1)	2·9 (1·2)	2·8 (1·2)	2·7 (1·0)	2·7 (1·0)	2·8 (1·1)	2·8 (1·1)

Data are mean (SD) or n (%) unless otherwise stated. SF-12=12-item Short-Form Health Survey.

A consistent pattern of worsening mental health was observed in the difference-in-difference analysis, both before and after adjustment for covariates (table 2). In the adjusted model, the MCS component of SF-12 decreased in intervention group 1 by 1·39 (95% CI −1·29 to 4·08; p=0·31) compared with control group 1 and by 2·29 (0·00 to 4·57; p=0·050) compared with control group 2. For intervention group 2, MCS scores in the adjusted model decreased by 2·45 (−0·57 to 5·48; p=0·11) compared with control group 1 and by 1·28 (−1·45 to 4·00; p=0·36) compared with control group 2. When pooling the data across both interventions, the change in MCS scores in the adjusted model was −2·13 (−4·17 to −0·10; p=0·040) compared with control group 1 and −2·21 (−4·13 to −0·30; p=0·024) compared with control group 2.

**Table 2:** Difference-in-difference estimates of the effects of Lone Parent Obligations on mental, physical, and self-rated health

	Model 1*		**Model 2**†	
	Control group 1	Control group 2	Control group 1	Control group 2
**Intervention group 1**				
Mental health	−1·41 (−4·10 to 1·28); 0·30	−2·30 (−4·58 to −0·02); 0·048	−1·39 (−4·08 to 1·29); 0·31	−2·29 (−4·57 to 0·00); 0·050
Physical health	−0·03 (−2·17 to 2·12); 0·98	0·31 (−1·46 to 2·09); 0·73	−0·04 (−2·19 to 2·10); 0·97	0·24 (−1·55 to 2·04); 0·79
Self-rated health	0·05 (−0·17 to 0·27); 0·66	0·11 (−0·08 to 0·29); 0·26	0·05 (−0·17 to 0·27); 0·67	0·11 (−0·07 to 0·30); 0·22
**Intervention group 2**				
Mental health	−2·47 (−5·49 to 0·55); 0·11	−1·10 (−3·83 to 1·62); 0·43	−2·45 (−5·48 to 0·57); 0·11	−1·28 (−4·00 to 1·45); 0·36
Physical health	0·63 (−1·79 to 3·04); 0·61	−0·48 (−2·49 to 1·53); 0·64	0·65 (−1·76 to 3·05); 0·59	−0·48 (−2·50 to 1·54); 0·64
Self-rated health	0·03 (−0·19 to 0·25); 0·80	0·10 (−0·10 to 0·31); 0·34	0·03 (−0·19 to 0·25); 0·79	0·11 (−0·10 to 0·32); 0·30
**Pooled effect**				
Mental health	−2·13 (−4·17 to −0·09); 0·040	−2·12 (−3·96 to −0·28); 0·024	−2·13 (−4·17 to −0·10); 0·040	−2·21 (−4·13 to −0·30); 0·024
Physical health	0·41 (−1·25 to 2·07); 0·63	−0·03 (−1·44 to 1·38); 0·97	0·42 (−1·23 to 2·07); 0·61	−0·17 (−1·65 to 1·32); 0·83
Self-rated health	0·04 (−0·11 to 0·20); 0·58	0·10 (−0·04 to 0·23); 0·17	0·04 (−0·11 to 0·20); 0·58	0·13 (−0·01 to 0·27); 0·071

Data are effect (95% CI); p value.

* Unadjusted.

† Adjusted for mother's age, number of children, and highest educational attainment at baseline.

The secondary outcomes changed little in the intervention groups compared with the control groups (table 2). Scores on the Physical Health Summary of the SF-12 in intervention group 1 changed by −0·04 (95% CI −2·19 to 2·10; p=0·97) compared with control group 1 and by 0·24 (−1·55 to 2·04; p=0·79) compared with control group 2. Self-rated health in intervention group 1 changed by 0·05 (−0·17 to 0·27; p=0·67) compared with control group 1 and by 0·11 (−0·07 to 0·30; p=0·22) compared with control group 2. The analyses of intervention group 2 and the pooled analyses also showed no clear effect.

We found no clear difference in the effect of new exposure to LPO by educational background (appendix). The findings of the robustness analyses were consistent with the findings of the primary and secondary analyses (appendix).

## Discussion

Our results suggest that requiring lone parents with school-age children to seek work as a condition of receiving welfare benefits might adversely affect their mental health. Although the effect size is modest at the individual level, the majority of lone parents receiving Income Support, of whom there were 737 000 in May, 2008,^18^ are exposed to the requirement, so the effect on population health might be substantial. We estimated an intention-to-treat effect, which is directly relevant to a policy maker's perspective, but which probably underestimates the effect on individuals who are directly affected by LPO. For example, some people considered as exposed to the intervention on the basis of their circumstances when they were interviewed before the policy change might have been exempt from LPO because of having a new child by the time of their follow-up interview.

Previous research^19^, ^20^, ^21^, ^22^ has suggested that welfare reforms might have adverse effects on the health of people with long-term sickness, elderly people, and the general population, but less evidence is available for lone parents. A 2017 Cochrane review^9^ included 12 randomised controlled trials of welfare-to-work interventions for lone parents, of which seven investigated mandatory interventions (all were in the USA). The review found that the small positive effects of the interventions on income and employment disappeared over time, often because control group participants found work independently. Effects on physical and mental health, although largely positive, were unlikely to be clinically significant, and the risk of depression remained very high in all groups.

Observational studies^23^ done in the years after the US welfare reform found mixed effects on health. More recent difference-in-difference studies with longer-term follow-ups reported small but consistently negative associations with depressive symptoms,^24^ days of good mental health and health behaviours,^25^ and self-reported health and disability.^26^ Such negative effects might occur for several reasons. In the USA, although lone-parent employment increased after the welfare reform, the incomes of the poorest lone mothers decreased substantially.^27^ One study^28^ found that extreme poverty increased by 68% after the welfare reform, and in 2010, 25% of lone parents with low income were known to be disconnected—ie, without any apparent means of support from wages or means-tested benefits.^29^ Furthermore, job quality was poor for many individuals who gained employment.^30^

Qualitative research, which has also found mainly negative effects, helps to shed light on the mechanisms through which conditionality affects health. A systematic review^31^ of such mechanisms identified in qualitative studies of mandatory work requirements suggested that lone parents in Australia, Canada, New Zealand, the UK, and the USA reported increased depression, stress, anxiety, and fatigue, which they attributed to the difficulties of combining lone-parent child-rearing with fulfilling work requirements. Loss of control over, and conflict between, different areas of life emerged as mechanisms connecting work requirements with poorer health.^31^ The type of employment gained was often short term, insecure, and poorly paid. Loss of income due to sanctions could lead to financial strain and food insufficiency. Another qualitative study^32^ in the UK also reported that benefit sanctions were linked to severe health effects due to insufficient nutrition. Altogether, the available evidence suggests that the introduction of mandatory employment requirements for lone parents is associated with negative effects on health. Although social security systems in different national settings have varying levels of stringency, it seems that imposing stricter job-seeking requirements might have negative health effects regardless of the prereform starting point.

In the context of continuing intensification of employment requirements for lone parents, these findings are particularly noteworthy. Universal Credit, implemented in the UK between 2016 and 2018 as a replacement for Income Support, is the most recent reform to the social security system to be implemented in the UK and involves a radical shift in the principles underlying such social transfers.^33^ In particular, conditionality now applies to working people on low incomes, including lone parents who will continue to be subject to work requirements until they are working for a minimum of 25 h per week. Furthermore, the value of in-work supplements has decreased substantially, which might undermine the potential mechanism through which work might improve health.

This study has several important strengths. First, we took a natural experimental approach, using policy changes to define intervention and control groups and difference-in-difference analyses to account for differences in observed and unobserved fixed characteristics across the groups. Together with our focus on intention-to-treat effects, this approach should have minimised the risk of confounding due to selective exposure to the intervention. Second, we explored the consistency of findings across a range of predefined intervention and control groups.^34^, ^35^ Lastly, we assessed the robustness of our findings to alternative modelling approaches and used multiple imputation to reduce the effect of missing data.

The following limitations should be noted. First, we cannot rule out the possibility of unobserved time-varying confounding, although the consistent findings from the analyses using control groups of lone mothers with children older and younger than our intervention groups make time-varying confounding unlikely. Second, although we used the UK's largest longitudinal panel study, the sample size available for analysis was small, and so the effects estimated were imprecise. The analysis to detect a differential effect by educational background had particularly low power. Third, as with all panel surveys, Understanding Society is subject to missing data and attrition. We attempted to minimise the effect of missing data and attrition by use of multiple imputation, applying inverse probability weights, and by doing a range of robustness checks. Fourth, we assessed only the short-term effects of the policy; the long-term effects remain unknown. Fifth, our analyses were restricted to lone parents receiving Income Support; conditionality might have also affected lone parents who were in work but believed they were likely to be exposed to the requirements in the future.

In conclusion, our analyses suggest that mandatory work requirements might have adverse effects on the mental health of lone parents in the short term. Priorities for future research should be to test whether these effects persist over time, whether the health of children in single-parent families is also affected, and whether similar effects occurred after the reduction in the age threshold from 5 to 3 years in 2017. Longer-term follow-up of families exposed to the requirements might make it possible to identify the key mechanisms underlying their effect on health, and therefore develop mitigating measures. The effects of further changes to the UK social security system, including the extension of conditionality to those who are already in work and the reduction in value of in-work income supplements, are also important areas for future research.
