# Supplementary material for: Effects of restrictions to Income Support on health of lone mothers in the UK: a natural experiment study
Source: Lancet Public Health. 2018 Jul 2;3(7):e333–40. doi: 10.1016/S2468-2667(18)30109-9 (PMC6038023; doi:10.1016/S2468-2667(18)30109-9)

THE LANCET
Public Health

Supplementary appendix

This appendix formed part of the original submission and has been peer reviewed. We post it as supplied by the authors.

Supplement to: Katikireddi SV, Molaodi OR, Gibson M, Dundas R, Craig P. Effects of restrictions to Income Support on health of lone mothers in the UK: a natural experiment study. *Lancet Public Health* 2018; **3:** e333–40.

Web Appendix

Statistical analysis

We fitted individual fixed effects linear regression models including an interaction term between follow-up and intervention group:

$$Y_{it} = \alpha_i + \beta_1 Int_i + \beta_2 follow_i + \beta_3 Age_i + \beta_4 Kids_i + \beta_5 Education_i + \beta_6\, follow_i * Int_i + \varepsilon_{it}$$

where Y is the outcome, i refers to each participant and t each observation time-point; Int is a dummy variable indicating receipt of intervention; Age is the maternal age at baseline; Kids is number of children; Education is the highest educational attainment; follow is a dummy variable indicating observations taken post intervention; follow*Int is the DiD estimator; and ε is the error term.

Web Table 1: Detailed characteristics of study participants for total sample and complete cases

	Total sample		Complete cases	
	**Baseline**	**Follow-up**	**Baseline**	**Follow-up**
**Intervention 1 (7 to 5)**	***N= 175***	***N=145***	***N=94***	***N=94***
Maternal age at baseline, Mean (SD)	33.0 (6.9)	33.2(7.1)	33.6 (6.6)	34.7 (6.7)
No of children (1), N (%)	75 (42.9)	61 (42.1)	40 (42.6)	38 (40.4)
2	64 (36.6)	54 (37.2)	36 (38.3)	35 (37.2)
3+	36 (20.6)	30 (20.7)	18 (19.2)	21 (22.3)
Highest education (Low), N (%)	118 (75.2)	94 (72.3)	66 (70.2)	66 (70.2)
Intermediate	11 (7.0)	11 (8.5)	9 (9.6)	9 (9.6)
High	28 (17.8)	25 (19.2)	19 (20.2)	19 (20.2)
SF 12 PCS, Mean (SD)	50.2 (11.3)	49.9 (11.0)	50.0 (10.8)	49.8 (10.0)
SF 12 MCS, Mean (SD)	44.6 (12.0)	44.4(11.9)	43.5 (12.0)	41.1 (12.4)
Self-rated health, Mean (SD)	2.8 (1.1)	2.8 (1.1)	2.8 (1.1)	3.0 (1.1)
**Control 1**	***N= 328***	***N=293***	***N=187***	***N=187***
Maternal age at baseline, Mean (SD)	27.5 (6.9)	27.7 (6.9)	27.6 (6.9)	28.7 (6.8)
No of children (1), N (%)	120 (36.6)	103 (35.2)	67 (35.8)	65 (34.8)
2	120 (36.6)	111 (37.9)	72 (38.5)	72 (38.5)
3+	88 (26.8)	79 (27.0)	48 (25.7)	50 (26.7)
Highest education (Low), N (%)	219 (76.0)	192 (75.3)	134 (71.2)	134 (71.2)
Intermediate	23 (8.0)	20 (7.8)	17 (9.1)	17 (9.1)
High	46 (16.0)	43 (16.9)	36 (19.3)	36 (19.3)
SF 12 PCS, Mean (SD)	52.02 (8.61)	51.9 (8.6)	51.8 (8.7)	51.6 (9.4)
SF 12 MCS, Mean (SD)	45.46 (11.35)	45.8 (11.2)	46.0 (10.2)	44.7 (11.6)
Self-rated health, Mean (SD)	2.66 (0.98)	2.6 (0.98)	2.6 (0.98)	2.8 (1.1)
**Control 2**	***N=944***	***N=835***	***N=571***	***N=571***
Maternal age at baseline, Mean (SD)	40.2 (6.99)	40.2 (6.9)	40.1 (6.7)	41.2 (6.7)
No of children (1), N (%)	555 (58.8)	489 (58.6)	331 (58.0)	367 (64.3)
2	304 (32.2)	273 (32.7)	192 (33.6)	165 (28.9)
3+	85 (9.00)	73 (8.7)	48 (8.4)	39 (6.8)
Highest education (Low), N (%)	488 (58.4)	431 (58.4)	315 (55.2)	315 (55.2)
Intermediate	66 (7.9)	61 (8.3)	47 (8.2)	47 (8.2)
High	282 (33.7)	246 (33.3)	209 (36.6)	209 (36.6)
SF 12 PCS, Mean (SD)	51.1 (10.6)	51.1 (10.6)	51.4 (10.4)	45.5 (11.6)
SF 12 MCS, Mean (SD)	45.2 (11.7)	45.0 (11.7)	45.5 (11.6)	45.2 (11.5)
Self-rated health, Mean (SD)	2.6 (1.1)	2.6 (1.1)	2.6 (1.1)	2.7 (1.1)

	Total sample		Complete cases	
	**Baseline**	**Follow-up**	**Baseline**	**Follow-up**
**Intervention 2 (10 to 7)**	***N=192***	***N=146***	***N=83***	***N=83***
Maternal age at baseline, Mean (SD)	354 (7.0)	35.1 (7.1)	35.2 (7.0)	36.2 (7.0)
No of children (1), N (%)	75 (39.1)	53 (36.3)	32 (38.6)	30 (36.1)
2	83 (43.2)	65 (44.5)	35 (42.2)	37 (44.6)
3+	34 (17.7)	28 (19.2)	16 (19.3)	16 (19.3)
Highest education (Low), N (%)	134 (75.7)	98 (74.8)	61 (73.5)	61 (73.5)
Intermediate	17 (9.6)	12 (9.2)	10 (12.1)	10 (12.1)
High	26 (14.7)	21 (16.0)	12 (14.5)	12 (14.5)
SF 12 PCS, Mean (SD)	50.3 (10.3)	50.7 (9.8)	51.8 (9.2)	50.9 (10.9)
SF 12 MCS, Mean (SD)	42.9 (12.5)	43.0 (12.3)	45.6 (11.2)	41.9 (14.9)
Self-rated health, Mean (SD)	2.9 (.2)	2.9 (1.2)	2.9 (1.2)	2.8 (1.2)
**Control 1**	***N=530***	***N=418***	***N=252***	***N=252***
Maternal age at baseline, Mean (SD)	28.6 (7.1)	28.8 (7.1)	29.1 (7.1)	30.1 (7.1)
No of children (1), N (%)	188 (35.5)	154 (36.8)	90 (35.2)	84 (33.3)
2	211 (39.8)	164 (39.2)	96 (38.1)	105 (41.7)
3+	131 (24.7)	100 (23.4)	66 (26.2)	63 (25.0)
Highest education (Low), N (%)	401 (81.3)	306 (79.5)	201 (79.8)	201 (79.8)
Intermediate	34 (6.9)	24 (6.2)	13 (5.2)	13 (5.2)
High	58 (11.8)	55 (14.3)	38 (15.1)	38 (15.1)
SF 12 MCS, Mean (SD)	52.6 (9.2)	52.3 (9.0)	52.3 (9.3)	50.9 (9.4)
SF 12 PCS, Mean (SD)	45.0 (11.9)	45.3 (11.7)	45.0 (12.2)	46.4 (11.0)
Self-rated health, Mean (SD)	2.7 (1.0)	2.7 (1.0)	2.7 (1.0)	2.7 (1.0)
**Control 2**	***N=623***	***N=522***	***N=284***	***N=284***
Maternal age at baseline, Mean (SD)	42.2 (6.0)	41.8 (6.0)	41.8 (5.9)	42.8 (5.9)
No of children (1), N (%)	418 (67.1)	259 (61.7)	174 (61.3)	203 (71.5)
2	181 (29.5)	141 (33.6)	99 (34.9)	71 (25.0)
3+	24 (3.9)	20 (4.8)	11 (3.9)	10 (3.5)
Highest education (Low), N (%)	328 (58.8)	198 (52.9)	136 (48.9)	136 (48.9)
Intermediate	59 (10.6)	44 (11.8)	35 (12.3)	35 (12.3)
High	171 (30.7)	132 (35.3)	113 (39.8)	113 (39.8)
SF 12 PCS, Mean (SD)	49.6 (11.4)	49.9 (11.5)	50.2 (11.2)	50.0 (11.2)
SF 12 MCS, Mean (SD)	45.8 (11.7)	45.9 (11.4)	46.5 (10.9)	46.2 (11.4)
Self-rated health, Mean (SD)	2.8 (1.1)	2.8 (1.1)	2.8 (1.1)	2.8 (1.1)

Web Table 2: Complete Case Analyses (with and without longitudinal weights)

	Complete case analyses with no weights			Complete case analyses with longitudinal weights		
	Effect size	95% CI	p value	Effect size	95% CI	p value
**Intervention 1 (7 to 5)**						
Mental health						
Effect compared to CG 1	-1.03	-3.98, 1.92	0.491	-0.31	-3.97, 3.34	0.867
Effect compared to CG 2	-2.14	-4.83, 0.55	0.119	-1.37	-4.70, 1.96	0.419
Physical health						
Effect compared to CG 1	0.06	-2.37, 2.49	0.959	-0.42	-3.17, 2.32	0.763
Effect compared to CG 2	0.16	-1.94, 2.25	0.883	0.26	-2.11, 2.62	0.831
Self-rated health						
Effect compared to CG 1	0.05	-0.17, 0.28	0.635	-0.04	-0.31, 0.22	0.759
Effect compared to CG 2	0.16	-0.03, 0.36	0.102	0.18	-0.05, 0.41	0.132
**Intervention 2 (10 to 7)**						
Mental health						
Effect compared to CG 1	-5.11	-8.49, -1.73	0.003	-5.05	-8.46, -1.63	0.004
Effect compared to CG 2	-3.75	-7.02, -0.48	0.025	-3.59	-6.90, -0.28	0.034
Physical health						
Effect compared to CG 1	0.49	-1.89, 2.88	0.685	0.21	-2.11, 2.52	0.86
Effect compared to CG 2	-0.98	-3.30, 1.35	0.409	-1.21	-3.42, 1.00	0.283
Self-rated health						
Effect compared to CG 1	-0.05	-0.28, 0.18	0.656	0.01	-0.25, 0.28	0.927
Effect compared to CG 2	0.04	-0.18, 0.27	0.702	0.14	-0.11, 0.39	0.279
**Pooled effect**						
Mental health						
Effect compared to CG 1	-2.61	-4.98, -0.24	0.031	-3.04	-5.53, -0.56	0.017
Effect compared to CG 2	-2.81	-4.89, -0.73	0.008	-2.36	-4.70, -0.02	0.048
Physical health						
Effect compared to CG 1	0.25	-1.54, 2.03	0.787	0.01	-1.75, 1.77	0.99
Effect compared to CG 2	-0.33	-1.88, 1.22	0.677	-0.45	-2.07, 1.18	0.59
Self-rated health						
Effect compared to CG 1	0.017	-0.15, 0.18	0.840	0.00	-0.18, 0.19	0.968
Effect compared to CG 2	0.147	-0.01, 0.31	0.072	0.18	-0.01, 0.37	0.06

Web Table 3: Analyses of all single parents (including males), multiply imputed

	**Effect size**	**95% CI**	**p value**
**Intervention 1** (7 to 5)			
Mental health			
Effect compared to CG 1	-1.58	-4.21, 1.04	0.236
Effect compared to CG 2	-2.44	-4.69, -0.19	0.034
Physical health			
Effect compared to CG 1	0.10	-2.00, 2.21	0.922
Effect compared to CG 2	0.36	-1.38, 2.11	0.683
Self-rated health			
Effect compared to CG 1	0.04	-0.18, 0.25	0.729
Effect compared to CG 2	0.11	-0.07, 0.29	0.233
**Intervention 2** (10 to 7)			
Mental health			
Effect compared to CG 1	-2.61	-5.57, 0.36	0.085
Effect compared to CG 2	-1.40	-4.05, 1.26	0.301
Physical health			
Effect compared to CG 1	0.82	-1.56, 3.19	0.497
Effect compared to CG 2	-0.48	-2.46, 1.51	0.638
Self-rated health			
Effect compared to CG 1	0.01	-0.21, 0.23	0.921
Effect compared to CG 2	0.09	-0.12, 0.29	0.405
**Pooled effect**			
Mental health			
Effect compared to CG 1	-2.32	-4.31, -0.32	0.023
Effect compared to CG 2	-2.31	-4.17, -0.45	0.015
Physical health			
Effect compared to CG 1	0.59	-1.05, 2.22	0.479
Effect compared to CG 2	-0.01	-1.47, 1.44	0.987
Self-rated health			
Effect compared to CG 1	0.03	-0.12, 0.18	0.706
Effect compared to CG 2	0.12	-0.02, 0.25	0.103

Web Table 4: Difference-in-difference estimates of the impact of lone parent obligations on mental, physical and self-rated health: intervention groups 1 and 2 compared to control group 1 (excluding parents whose youngest child was less than one year old), multiply imputed

	Effect size	95% CI	p value
**Intervention 1** (7 to 5)			
Mental health	-1.87	-4.63, 0.89	0.183
Physical health	0.39	-1.88, 2.67	0.733
Self-rated health	0.05	-0.19, 0.28	0.700
**Intervention 2** (10 to 7)			
Mental health	-2.45	-5.52, 0.62	0.117
Physical health	0.65	-1.79, 3.09	0.601
Self-rated health	0.06	-0.17, 0.28	0.617
**Pooled Effect**			
Mental health	-2.34	-4.43, -0.26	0.028
Physical health	0.60	-1.10, 2.29	0.488
Self-rated health	0.06	-0.10, 0.22	0.460

Web Table 5: Difference-in-differences models for maternal demographic characteristics (pooled analysis), complete case analysis

	Odds ratio	95% CI	p value
**Control 1**			
Maternal Age	-0.057*	-0.26, 1.49	0.588
Intermediate (vs low education)	1.011	0.96, 1.06	0.660
Tertiary (vs low education)	1.00	0.97, 1.04	0.774
**Control 2**			
Maternal Age	0.019*	-0.082, 0.12	0.647
Intermediate (vs low education)	0.963	0.92, 1.01	0.136
Tertiary (vs low education)	0.991	0.95, 1.03	0.649

*Effect size

Web Table 6: Difference-in-difference estimates of the impact of lone parent obligations on mental, physical and self-rated health (pooled analysis), multiply imputed

	Model 1	95% CI	p value	Model 2	95% CI	p value
Mental health						
Effect compared to control group 2*	-2.33	-4.33, -0.33	0.022	-2.43	-4.50, -0.36	0.022
Physical health						
Effect compared to control group 2*	0.079	-1.45, 1.61	0.568	-0-053	-1.66, 1.56	0.949
Self-rated health						
Effect compared to control group 2*	0.12	-0.026, 0.27	0.105	0.15	-0.008, 0.30	0.063

*Restricted to mothers whose youngest child was aged 7-9 years (intervention 1) and 10-12 years (intervention 2). Model 1 unadjusted and Model 2 adjusted for maternal age, education and number of children.

Web Table 7: Difference-in-difference estimates of the impact of lone parent obligations on mental, physical and self-rated health using dummies for maternal age (20-29, 30+) and age of youngest child (1, 2, 3+) (pooled analysis), multiply imputed

	Effect size	95% CI	p value
Mental health			
Effect compared to control group 1	-2.18	-4.23, -0.14	0.036
Effect compared to control group 2	-2.23	-4.15, 0.31	0.023
Physical health			
Effect compared to control group 1	0.38	-1.28, 2.04	0.65
Effect compared to control group 2	-0.16	-1.64, 1.38	0.837
Self-rated health			
Effect compared to control group 1	0.048	-0.11, 0.20	0.54
Effect compared to control group 2	0.13	-0.011, 0.27	0.072

Web Table 8: Difference-in-difference estimates of the impact of lone parent obligations on inequalities in mental, physical and self-rated health effect by educational attainment. Model adjusted for maternal age and number of children, (pooled analysis), multiply imputed

	**Effect size**	**95% CI**	**p value**
Mental health			
Effect compared to control group 1	1.59	-3.23, 6.42	0.516
Effect compared to control group 2	1.80	-2.66, 6.25	0.429
Physical health			
Effect compared to control group 1	-2.37	-5.77, 1.02	0.170
Effect compared to control group 2	-2.92	-6.18, 0.33	0.078
Self-rated health			
Effect compared to control group 1	0.22	-0.14, 0.58	0.228
Effect compared to control group 2	0.38	0.058, 0.70	0.021

Web Figure 1: Mean SF12 mental component scores for intervention group 1 and control groups 1 and 2

(a) Complete cases

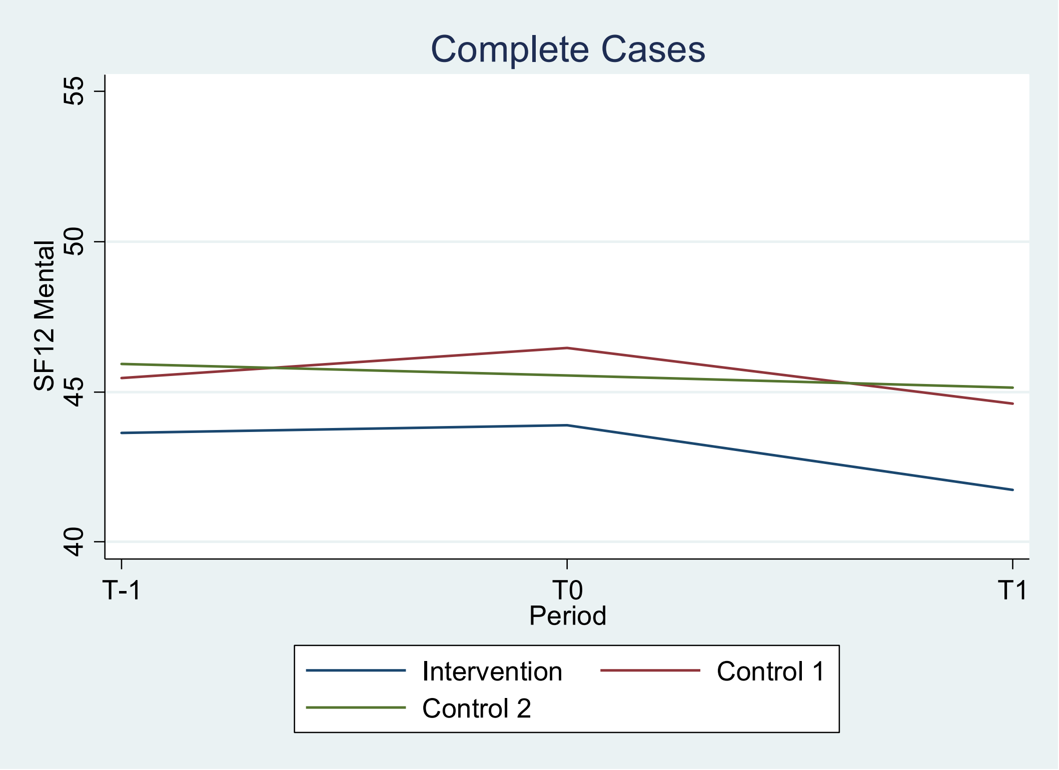

(b) All cases

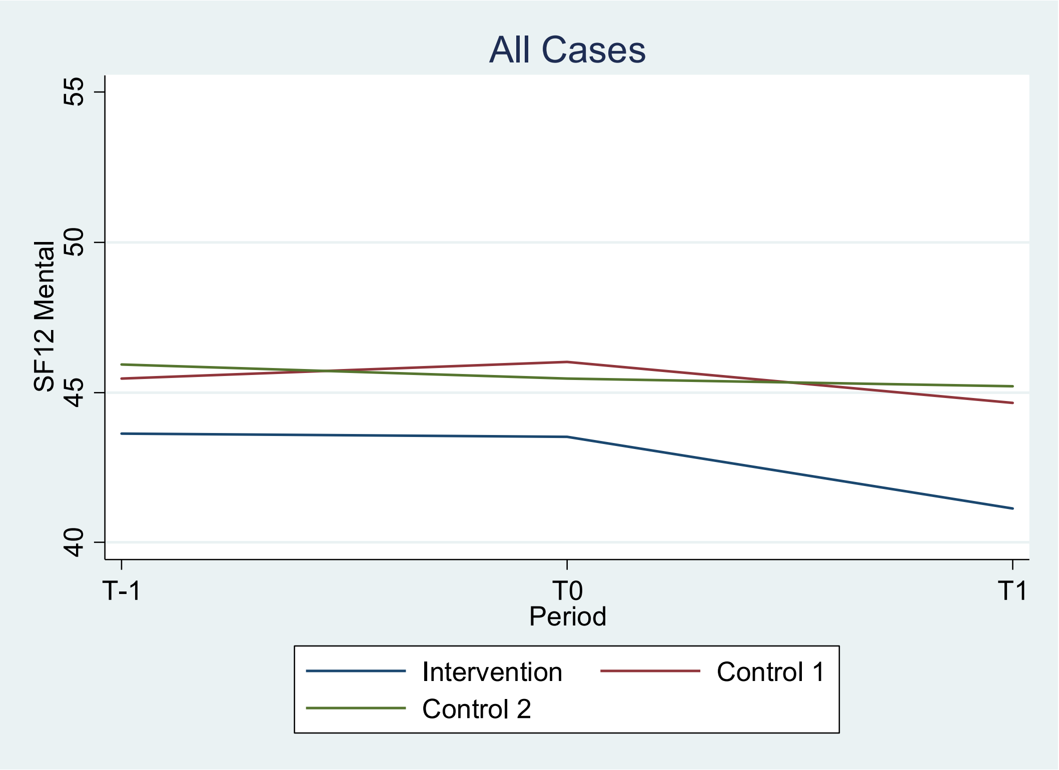

Web Figure 2: Mean SF12 physical component scores for intervention group 1 and control groups 1 and 2

(a) Complete cases

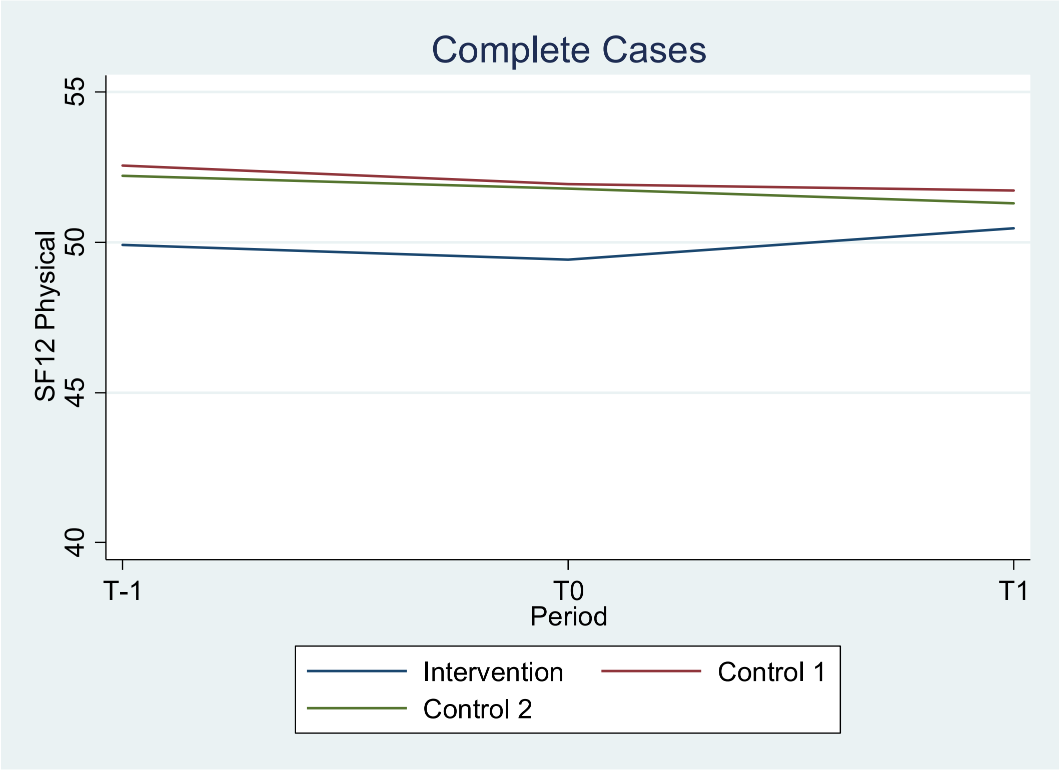

(b) All cases

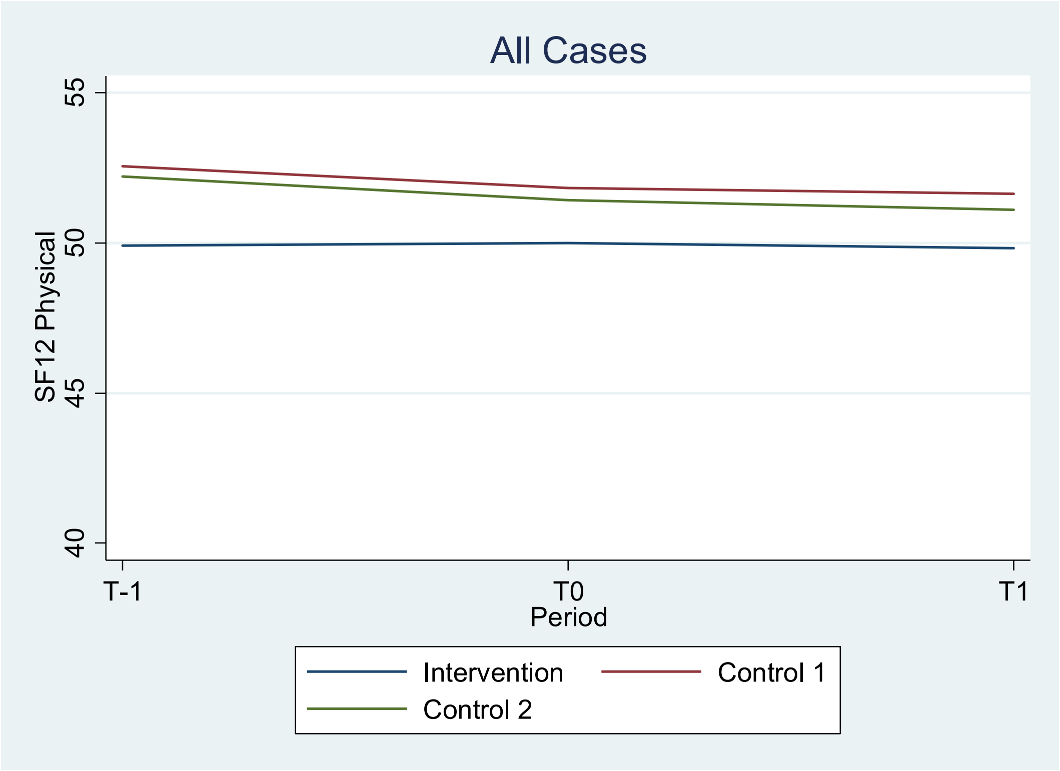

Supplement: Supplementary appendix [file mmc1.pdf]
